# Supplementary figures and images for: Spatiotemporal changes in river network connectivity in the Nile River Basin due to hydropower dams
Source: PLoS One. 2025 Apr 29;20(4):e0322338. doi: 10.1371/journal.pone.0322338 (PMC12040129; doi:10.1371/journal.pone.0322338)

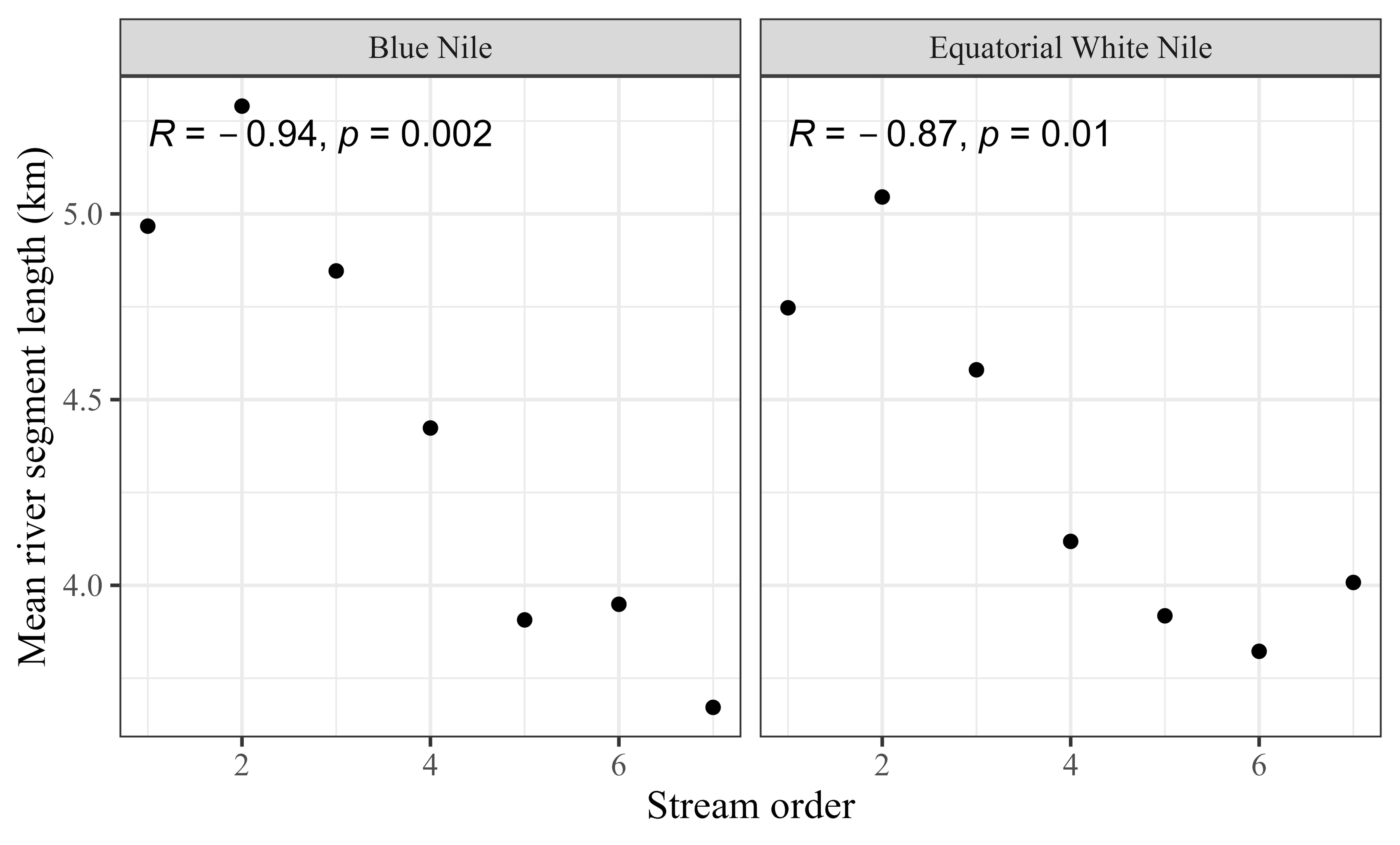

Supplement: Fig S1 — (TIF) [file pone.0322338.s001.tif]
